# Yeast pre-rRNA is processed at the A' site

**DOI:** 10.17912/micropub.biology.001593

**Published:** 2025-06-27

**Authors:** Laura M. Dutca, Emily F. Freed, Susan J. Baserga

**Affiliations:** 1 Molecular Biophysics & Biochemistry, Yale University, New Haven, Connecticut, Iowa City VA Health Care System, Iowa City, Iowa, United States; 2 Genetics, Yale School of Medicine, New Haven, Connecticut, Dept. of Physics, Colorado School of Mines, Golden, Colorado, United States; 3 Molecular Biophysics & Biochemistry, Genetics and Therapeutic Radiology, Yale University and the Yale School of Medicine, New Haven, Connecticut, United States

## Abstract

Maturation of the pre-ribosomal RNA (pre-rRNA) in eukaryotes involves a series of processing steps that remove transcribed spacer sequences to produce mature ribosomes. In many organisms two processing sites are located in the 5’external transcribed spacer (5’ETS), along with the site that defines the 5’-end of the 18S rRNA. However, the pre-rRNA processing site near the start site of transcription, known as A’, has long been believed to be absent in the single-celled yeast, *Saccharomyces cerevisiae* . Here we provide evidence that the A’ pre-rRNA processing site is also present in the yeast 5’ETS, confirming conservation among single-celled and multicellular eukaryotes.

**Figure 1. Yeast pre-rRNA is processed at the A' site:**
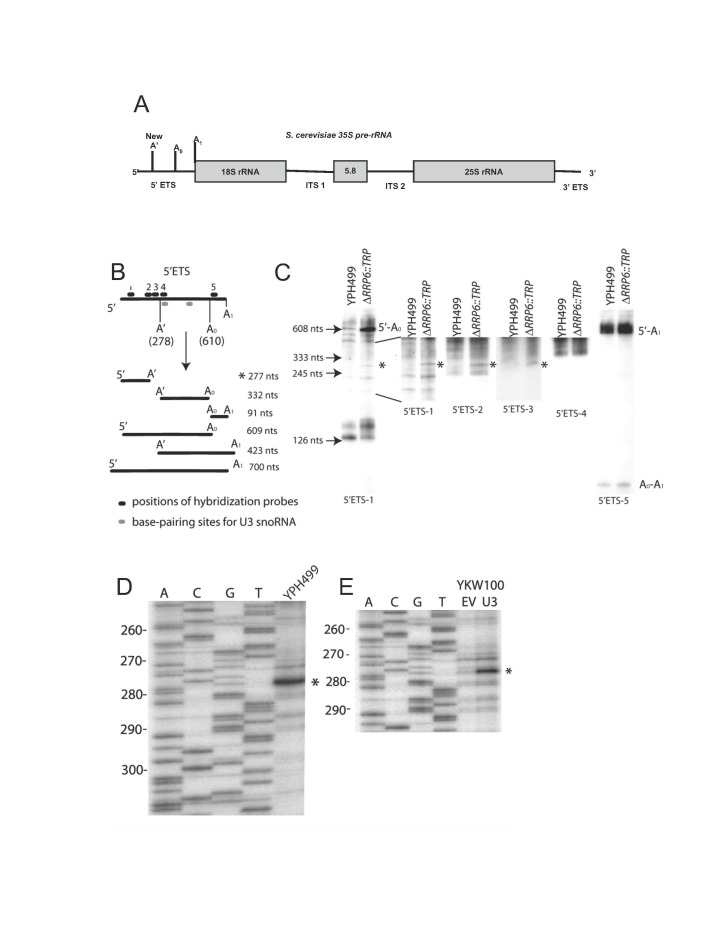
**Figure 1A** . Schematic representation of the 35S pre-rRNA in *S. cerevisiae* . The 35S pre-rRNA encodes external and internal transcribed spacers (5’ETS, 3’ETS and ITS1, ITS2), which are processed to make the mature 18S, 5.8S and 25S rRNAs. Only the pre-rRNA processing sites in the 5’ETS are indicated, with A’ specified as “New”. **Figure 1B.** Predicted pre-rRNA cleavage fragments from the yeast 5’ETS. Schematic representation of the 5’ETS with the three processing sites (A’, A _0_ , A _1_ ) and the predicted RNA fragments generated by processing at these sites. The annealing positions of the oligonucleotide probes (5’ETS-1 to 5) are depicted above the pre-rRNA. The U3 snoRNA binding sites are shown in gray below the pre-rRNA. The asterisk indicates the 5’-A’ rRNA fragment resulting from processing at A’. **Figure 1C** . The A’ processing site in *S. cerevisiae* is detectable by northern blotting of pre-rRNA processing intermediates in the Δ *rrp6::TRP* strain. Northern blot analysis of pre-rRNA fragments from the 5’ ETS with the indicated oligonucleotides probes (5’ETS-1 to 5) from Fig. 1B. Total RNA was extracted from the parental YPH499 and the Δ *rrp6::TRP* strains and analyzed on 8% polyacrylamide gels. As markers, the U14 snoRNA (126 nts), snR10 (245 nts), U3 snoRNA (333 nts) and snR30 (608 nts) were also probed for with complementary oligonucleotides (arrows). The asterisk indicates the 5’-A’ rRNA fragment resulting from processing at A’. The pre-RNA fragments of length about 130 nts visible with the 5'ETS-1 probe are consistently present in northern blots, but their identity has not been established. **Figure 1D** . Processing at the A’ site can be detected by primer extension. Total RNA was extracted from the parental YPH499 strain and analyzed by primer extension with an oligonucleotide complementary to nts 401-423 (lane YPH499). The A’ cleavage site is indicated by an asterisk. Lanes A,C,G,T represent a sequencing ladder. **Figure 1E** . Processing at the A’ site is U3 snoRNA-dependent. Strain YKW100, either bearing empty vector (EV) or a plasmid constitutively expressing the U3 snoRNA (U3), was grown in glucose to repress expression of the genomic U3 snoRNA. Total RNA was extracted and analyzed by primer extension using an oligonucleotide complementary to nts 401-423. The A’ cleavage site is indicated by an asterisk. Lanes A,C,G,T represent a sequencing ladder.

## Description

Ribosome biogenesis, the process of making ribosomes, occurs in the nucleolus in all eukaryotes. It is a complex and energy-intensive process (Warner, 1999) that requires the activity of all 3 RNA polymerases (Aubert et al., 2018; Bohnsack & Bohnsack, 2019; Dörner et al., 2023; Tomecki et al., 2017). The process begins with the transcription of the primary transcript, the 35S pre-rRNA in *Saccharomyces cerevisiae* ( *S. cerevisiae* ; Fig. 1A ), by RNA polymerase I. Pre-rRNA transcription is followed by a series of pre-rRNA processing steps that produce the 18S rRNA (small ribosomal subunit) and the 5.8S and 25S rRNAs (large ribosomal subunit) which are assembled with ribosomal proteins to make mature ribosomes (Vanden Broeck & Klinge, 2024). The U3 small ribonucleoprotein (U3 snoRNP), a key component of the SSU processome (Dragon et al., 2002), is essential for the pre-rRNA cleavages that produce the mature 18S rRNA (Hughes & Ares, 1991; Samarsky & Fournier, 1998; Wehner et al., 2002; Wormsley et al., 2001)

Among the many pre-18S rRNA processing sites identified in eukaryotes, the A’ site in the 5’ETS ( Fig. 1A ) is not thought to be found in the yeast *S. cerevisiae* . This site is referred to as A’ or 01 in humans, A’ or 0 in mouse, A’ in trypanosomes or P in *Arabidopsis thaliana* (Craig et al., 1987; Hartshorne & Toyofuku, 1999; Kass et al., 1987; Kent et al., 2009; Mullineux & Lafontaine, 2012; Rouquette et al., 2005; Sikorska et al., 2017; Tomecki et al., 2017). In yeast the presence of this site, referred to here as A’, has not been detected and is generally believed to be absent in this organism (Bohnsack & Bohnsack, 2019; Mullineux & Lafontaine, 2012; Tomecki et al., 2017; Venema & Tollervey, 1999;Woolford & Baserga, 2013).

To determine whether the A’ processing site exists in yeast, we analyzed pre-rRNA isolated from a strain in which the 3’ to 5’ exonuclease component of the exosome, *RRP6* , was disrupted ( *Δrrp6* ). Rrp6 plays a role in degrading pre-rRNA fragments and its absence allows the detection of previously unstable pre-rRNAs (Allmang et al., 1999; Kent et al., 2009; Kobyłecki et al., 2018; Sloan et al., 2014). Using northern blotting and primer extension, we confirm the presence of the A’ processing site in the 5’ETS of *S. cerevisiae* pre-rRNA when Rrp6 is absent. We further confirm the presence of A’ processing by primer extension in the parent strain (YPH499) and demonstrate that the presence of the U3 snoRNA is required for optimal processing at A’. These findings provide evidence for the conservation of this pre-rRNA processing site from the yeast, *S. cerevisiae* , to humans.

**Northern blots of pre-rRNA processing intermediates reveal A’ site cleavage during yeast ribosome biogenesis.** Disruption of *RRP6* ( *Δrrp6)* , a gene encoding a nuclear exosome component, has previously been used to detect short-lived pre-rRNA processing intermediates (Allmang et al., 1999; Kent et al., 2009; Kobyłecki et al., 2018; Sloan et al., 2014). In the *Δrrp6* strain, pre-rRNA processing intermediates or fragments accumulate, which facilitates their detection. To enhance the identification of A’ site processing, we generated the *Δrrp6* :: *TRP* strain in which the *RRP6* gene is disrupted. We analyzed the pre-rRNA fragments resulting from 5’ETS processing in this strain compared to the parent strain, YPH499, by northern blotting using oligonucleotide probes complementary to the 5’ ETS.

Northern blot analysis yields a pre-rRNA fragment that extends from the transcription start site (5’) to a new cleavage site (A’) when *RRP6* is disrupted. Figure 1B shows the predicted lengths of potential processing products of the 5’ETS if cleavage were also to occur at A’, as well as at the known A0 and A1 sites. We focused on fragments smaller than the 5’ETS (700 nts), since A’ processing is predicted to occur near the 5’ end of the 5’ETS. We used a series of oligonucleotide probes that anneal to different sequences in the 5’ETS to identify the pre-rRNAs (5’ ETS1-5 in Fig. 1B ). Hybridization to snoRNAs of known lengths was used to size the pre-rRNA fragments ( Figure 1C, numbered arrows). In the *Δrrp6* strain, changes in the levels of many of the pre-rRNA fragments are detectable ( Fig. 1C ). As expected, the 5’-A _0_ pre-rRNA fragment (608 nt) is the most prominent fragment to accumulate to a higher level in the *Δrrp6* strain when compared to the parent strain ( Fig. 1C, 5'ETS-1 left). The 5’-A _1_ pre-rRNA fragment (about 700 nts) is also visible ( Fig. 1C, 5'ETS-5). There is a new pre-rRNA fragment (5’-A’) in the *Δrrp6* strain, about 300 nt long, that hybridizes to probes 5’ to the A’ pre-rRNA cleavage site ( Fig. 1C, 5'ETS-1 right, 5'ETS-2, 5'ETS-3, asterisks), but not to probes 3’ to the A’ cleavage site ( Fig. 1C, 5'ETS-4, 5'ETS-5). This pre-rRNA fragment results from processing at a new site, A’, in the pre-rRNA, generating the 5’-A’ pre-rRNA fragment.

**The A’ pre-rRNA processing site is detectable by primer extension.** To precisely localize the A’ cleavage site in the YPH499 parent yeast strain, we performed primer extension on the pre-rRNA using a primer that anneals between the proposed A’ processing site and A _0_ at nucleotides (nts) 401-423 (see 400-5’ETS in Table 1). To precisely map the cleavage site, a sequencing ladder was run at the same time ( Fig. 1D, lanes A,C,G,T). The primer extension revealed a few weak stops and a strong stop at nt 278, which we interpret as processing between nts 277/278 ( Figure 1D lane YPH499). Thus, the A’ processing site is detectable at nt 278 by primer extension in *S. cerevisiae* .

**A’ processing depends on the presence of the U3 snoRNA** . Since the A _1_ and A _0_ cleavages in the 5’ETS are dependent on the presence of the U3 snoRNA (Hughes & Ares, 1991; Samarsky & Fournier, 1998; Wehner et al., 2002; Wormsley et al., 2001), we investigated whether cleavage at A’ is similarly dependent. We compared cleavage at nts 277/278 in the presence and absence of the U3 snoRNA by primer extension. RNA was extracted from the YKW100 strain, in which the only remaining U3 snoRNA gene is under a galactose inducible/ glucose repressible promoter (Wehner et al., 2002). In glucose, yeast can grow and process pre-18S rRNA when the U3 snoRNA is expressed from a plasmid, but not when carrying an empty vector (Hughes, 1996). We observed that the primer extension stop at nt 278 is severely reduced in yeast cells carrying the empty vector (EV) when grown in glucose, where the U3 snoRNA is not expressed ( Figure 1E lane EV). However, when the U3 snoRNA is expressed from a plasmid under the same conditions, the stop at nt 278 remains strong ( Figure 1E compare lanes EV and U3). These results demonstrate that efficient processing at the A’ site requires the presence of the U3 snoRNA.

Here we have shown that pre-rRNA processing in *S. cerevisiae* pre-rRNA also occurs at the A’ site in the 5’ETS, as reported for other eukaryotes such as humans, mice, plants and trypanosomes (Craig et al., 1987; Hartshorne & Toyofuku, 1999; Kass et al., 1987; Kent et al., 2009; Mullineux & Lafontaine, 2012; Rouquette et al., 2005; Sikorska et al., 2017; Tomecki et al., 2017). We have confirmed the presence of the cleavage site at nt 277/278 in the 5’ETS by northern blotting after gene disruption of the 3’ to 5’ exonuclease, *RRP6,* and by primer extension. Using genetic depletion, we show that processing at the A’ site depends on the presence of the U3 snoRNA, which base pairs at nt 282-292 (Dutca et al., 2011; Marmier-Gourrier et al., 2011). Thus, pre-rRNA processing and the role of the U3 snoRNA in mediating cleavage in the 5’ ETS are similar among many of the studied eukaryotes, including in unicellular organisms like *S. cerevisiae* and *T. brucei* .

Rrp6 is a 3’ to 5’ exoribonuclease that is present mainly in the nucleus and is associated with the RNA exosome (Fox & Mosley, 2016; Januszyk & Lima, 2014; Stuparević et al., 2021; Zinder & Lima, 2017). Our results show that in *S. cerevisiae* the protein Rrp6 participates in the degradation of the pre-rRNA fragments, 5’-A’ and 5’-A _0_ , resulting from the processing that takes place in the 5’ETS. These pre-rRNAs are more abundant or detectable only in the *Δrrp6* strain. Accumulation of the 5’-A _0_ fragment has also been observed in earlier studies in *S. cerevisiae* when components of the exosome or exosome associated factors were depleted (Allmang et al., 1999; de la Cruz et al., 1998). The Rrp6 homologs from humans and mice (EXOSC10) and *Arabidopsis thaliana* (RRP6L2) also participate in the degradation of 5’ETS rRNA fragments (Kent et al., 2009; Kobyłecki et al., 2018; Lange et al., 2008). Likewise, the role of the Rrp6 protein in ribosome biogenesis is similar in diverse eukaryotes.

## Methods

**Strains and media.** The *Δrrp6::TRP* strain was generated from the strain YPH499 ( *MAT* ***a*** *ura3-52 lys2-801 ade2-101 trp1-Δ63 his3-Δ200 leu2-Δ1* ) using the pFA6a-TRP1 plasmid as described (Longtine et al., 1998). The YKW100 ( *MAT* ***a*** *ura3-52 his3-Δleu2 lys2-801 ^amber^ trp1-Δ63 u3aΔ UAS _GAL_ :U3A::URA3 u3bΔ::LEU2* ) (Wehner et al., 2002) strain was used in primer extension experiments. Yeast were grown in YPD (1% yeast extract, 2% peptone, 2% dextrose), YPG/R (1% yeast extract, 2% peptone, 2% galactose and 2% raffinose) or yeast selective media (SC-TRP, Clontech) supplemented with either 2% dextrose or 2% galactose and 2% raffinose. The solid medium contained 2% Bactoagar. Yeast transformations were performed using the standard lithium acetate protocol (Gietz et al., 1995). For depletion experiments, the YKW100 strain with the appropriate plasmid was grown in selective media (SC-TRP, Clontech) with galactose and raffinose to an optical density at 600 nm of 0.4-0.8 and shifted to YPD for 16 hours.

**Plasmids.** The plasmid pRS314 U3 WT contains a copy of the U3 snoRNA gene in the yeast expression vector pRS314 ( *AMP ^R^ , TRP, CEN/ARS* ) (Wehner et al., 2002). After transforming the plasmids into YKW100, the strains were maintained on SC-TRP.

**RNA manipulations: primer extension and northern blots.** Total RNA was obtained by hot phenol extraction (Ausubel, 1995). For analyzing small RNAs, 7 mg of total RNA were separated on 8% denaturing polyacrylamide gels and transferred to Hybond XL membranes (GE Healthcare), as described (Samarsky & Fournier, 1998; Wormsley et al., 2001). A series of oligonucleotides were used to detect the desired pre-rRNAs and snoRNAs (see Figure 1 and Table 1).

The primer extension protocol was performed as described (Dutca et al., 2011) with the following modifications: 2 mg of total RNA were used with 1 pmol of a 5’end- ^32^ P labeled oligonucleotide. The oligonucleotides used are in Table 1. The primer extension reaction was performed at 45 ^o^ C.

## Reagents

**Table 1** . List of oligonucleotides used for generating the delta *rrp6* strain, northern blotting and primer extension.

**Table:** 

Oligonucleotide	Sequence
RRP6.F1	5’-TAGACGAAATAGGAACAACAAACAGCTTATAAGCAC CCAATAAGTGCGTTCGGATCCCCGGGTTAATTAA-3’
RRP6.R1	5’-ATGAAAATTACCATAATTTATAAATAAAAAAATACG CTTGTTTTACATAAGAATTCGAGCTCGTTTAA AC -3’
5’-ETS1	5’-GTCTTCAACTGCTTTCGCAT C-3’
400-5’ETS	5'-GGAATGGTACGTTTGATATCGCT-3'
5’-ETS2	5’- CCC ACG ATG AGACTGTTCAG-3’
5’-ETS3	5’-GCTCACCAATGGAATCGCAAG-3’
5’-ETS4	5’-GCTAGTAATCCACCAAATCCTTC-3’
5’ETS-5	5’-CCACCTATTCCCTCTTGCTAGAAG-3’

## References

[R1] Allmang C. (1999). Functions of the exosome in rRNA, snoRNA and snRNA synthesis. The EMBO Journal.

[R2] Aubert Maxime, O’Donohue Marie-Françoise, Lebaron Simon, Gleizes Pierre-Emmanuel (2018). Pre-Ribosomal RNA Processing in Human Cells: From Mechanisms to Congenital Diseases. Biomolecules.

[R3] Bohnsack Katherine E, Bohnsack Markus T (2019). Uncovering the assembly pathway of human ribosomes and its emerging links to disease. The EMBO Journal.

[R4] Craig N, Kass S, Sollner-Webb B (1987). Nucleotide sequence determining the first cleavage site in the processing of mouse precursor rRNA.. Proceedings of the National Academy of Sciences.

[R5] de la Cruz J. (1998). Dob1p (Mtr4p) is a putative ATP-dependent RNA helicase required for the 3' end formation of 5.8S rRNA in Saccharomyces cerevisiae. The EMBO Journal.

[R6] Dörner Kerstin, Ruggeri Chiara, Zemp Ivo, Kutay Ulrike (2023). Ribosome biogenesis factors—from names to functions. The EMBO Journal.

[R7] Dragon François, Gallagher Jennifer E. G., Compagnone-Post Patricia A., Mitchell Brianna M., Porwancher Kara A., Wehner Karen A., Wormsley Steven, Settlage Robert E., Shabanowitz Jeffrey, Osheim Yvonne, Beyer Ann L., Hunt Donald F., Baserga Susan J. (2002). A large nucleolar U3 ribonucleoprotein required for 18S ribosomal RNA biogenesis. Nature.

[R8] Dutca Laura M., Gallagher Jennifer E. G., Baserga Susan J. (2011). The initial U3 snoRNA:pre-rRNA base pairing interaction required for pre-18S rRNA folding revealed by in vivo chemical probing. Nucleic Acids Research.

[R9] Fox Melanie J., Mosley Amber L. (2015). Rrp6: Integrated roles in nuclear RNA metabolism and transcription termination. WIREs RNA.

[R10] Gietz R. Daniel, Schiestl Robert H., Willems Andrew R., Woods Robin A. (1995). Studies on the transformation of intact yeast cells by the LiAc/SS‐DNA/PEG procedure. Yeast.

[R11] Hartshorne T., Toyofuku W. (1999). Two 5'-ETS regions implicated in interactions with U3 snoRNA are required for small subunit rRNA maturation in Trypanosoma brucei. Nucleic Acids Research.

[R12] Howland J L (1996). Short protocols in molecular biology, third edition: Edited by F Ausubel, R Brent, R E Kingston, D D Moore, J G Seidman, J A Smith and K Struhl. P 836. John Wiley & Sons, New York. 1995. $74.95. ISBN 0‐471‐13781‐2. Biochemical Education.

[R13] Hughes John M.X. (1996). Functional Base-pairing Interaction Between Highly Conserved Elements of U3 Small Nucleolar RNA and the Small Ribosomal Subunit RNA. Journal of Molecular Biology.

[R14] Hughes J.M., Ares M. (1991). Depletion of U3 small nucleolar RNA inhibits cleavage in the 5′ external transcribed spacer of yeast pre-ribosomal RNA and impairs formation of 18S ribosomal RNA.. The EMBO Journal.

[R15] Januszyk Kurt, Lima Christopher D (2014). The eukaryotic RNA exosome. Current Opinion in Structural Biology.

[R16] Kass Susan, Craig Nessly, Sollner-Webb Barbara (1987). Primary Processing of Mammalian rRNA Involves two Adjacent Cleavages and is not Species Specific. Molecular and Cellular Biology.

[R17] Kent Tatyana, Lapik Yevgeniya R., Pestov Dimitri G. (2008). The 5′ external transcribed spacer in mouse ribosomal RNA contains two cleavage sites. RNA.

[R18] Kobyłecki Kamil, Drążkowska Karolina, Kuliński Tomasz M., Dziembowski Andrzej, Tomecki Rafał (2018). Elimination of 01/A′–A0 pre-rRNA processing by-product in human cells involves cooperative action of two nuclear exosome-associated nucleases: RRP6 and DIS3. RNA.

[R19] Lange Heike, Holec Sarah, Cognat Valérie, Pieuchot Laurent, Le Ret Monique, Canaday Jean, Gagliardi Dominique (2008). Degradation of a Polyadenylated rRNA Maturation By-Product Involves One of the Three RRP6-Like Proteins in
*Arabidopsis thaliana*. Molecular and Cellular Biology.

[R20] Longtine Mark S., Mckenzie III Amos, Demarini Douglas J., Shah Nirav G., Wach Achim, Brachat Arndt, Philippsen Peter, Pringle John R. (1998). Additional modules for versatile and economical PCR-based gene deletion and modification in Saccharomyces cerevisiae. Yeast.

[R21] Marmier-Gourrier Nathalie, Cléry Antoine, Schlotter Florence, Senty-Ségault Véronique, Branlant Christiane (2011). A second base pair interaction between U3 small nucleolar RNA and the 5′-ETS region is required for early cleavage of the yeast pre-ribosomal RNA. Nucleic Acids Research.

[R22] Mullineux Sahra-Taylor, Lafontaine Denis L.J. (2012). Mapping the cleavage sites on mammalian pre-rRNAs: Where do we stand?. Biochimie.

[R23] Rouquette Jacques, Choesmel Valérie, Gleizes Pierre-Emmanuel (2005). Nuclear export and cytoplasmic processing of precursors to the 40S ribosomal subunits in mammalian cells. The EMBO Journal.

[R24] Samarsky Dmitry A., Fournier Maurille J. (1998). Functional Mapping of the U3 Small Nucleolar RNA from the Yeast
*Saccharomyces cerevisiae*. Molecular and Cellular Biology.

[R25] Sikorska Natalia, Zuber Hélène, Gobert Anthony, Lange Heike, Gagliardi Dominique (2017). RNA degradation by the plant RNA exosome involves both phosphorolytic and hydrolytic activities. Nature Communications.

[R26] Sloan Katherine E., Bohnsack Markus T., Schneider Claudia, Watkins Nicholas J. (2014). The roles of SSU processome components and surveillance factors in the initial processing of human ribosomal RNA. RNA.

[R27] Stuparević Igor, Novačić Ana, Rahmouni A. Rachid, Fernandez Anne, Lamb Ned, Primig Michael (2021). Regulation of the conserved 3′‐5′ exoribonuclease EXOSC10/Rrp6 during cell division, development and cancer. Biological Reviews.

[R28] Tomecki Rafal, Sikorski Pawel J., Zakrzewska‐Placzek Monika (2017). Comparison of preribosomal RNA processing pathways in yeast, plant and human cells – focus on coordinated action of endo‐ and exoribonucleases. FEBS Letters.

[R29] Vanden Broeck Arnaud, Klinge Sebastian (2024). Eukaryotic Ribosome Assembly. Annual Review of Biochemistry.

[R30] Venema Jaap, Tollervey David (1999). Ribosome Synthesis in
*Saccharomyces cerevisiae*. Annual Review of Genetics.

[R31] Warner Jonathan R (1999). The economics of ribosome biosynthesis in yeast. Trends in Biochemical Sciences.

[R32] Wehner Karen A., Gallagher Jennifer E. G., Baserga Susan J. (2002). Components of an Interdependent Unit within the SSU Processome Regulate and Mediate Its Activity. Molecular and Cellular Biology.

[R33] Woolford John L, Baserga Susan J (2013). Ribosome Biogenesis in the Yeast
*Saccharomyces cerevisiae*. Genetics.

[R34] WORMSLEY STEVEN, SAMARSKY DMITRY A., FOURNIER MAURILLE J., BASERGA SUSAN J. (2001). An unexpected, conserved element of the U3 snoRNA is required for Mpp10p association. RNA.

[R35] Zinder John C., Lima Christopher D. (2017). Targeting RNA for processing or destruction by the eukaryotic RNA exosome and its cofactors. Genes & Development.

